# Left ventricle endomyocardial fibrosis: a case report

**DOI:** 10.1186/s13256-023-04056-z

**Published:** 2023-08-12

**Authors:** Raquel Reis Soares, Maria Clara Martins Avelar, Sofia Lucena Zanetti, Joao Victor Tavares Mendonça Garreto, Vinicius Dinelli Guimaraes, Elisa Soares Ferber, Mayumi de Oliveira Drumond, Matheus Ferber, Leonardo Ferber

**Affiliations:** 1 Biocor Rede D’Or Institute, Nova Lima, Brazil; 2 Faculdade Ciências Médicas de Minas Gerais, Belo Horizonte, Brazil; 3 Universidade José do Rosário Vellano – UNIFENAS, Alfenas, Brazil; 4 Universidade Federal dos Vales do Jequitinhonha e Mucuri, Teófilo Otoni, Brazil; 5 Hospital das Clinicas da Universidade Federal de Minas Gerais, Belo Horizonte, Brazil

**Keywords:** Restrictive cardiomyopathy, Endomyocardial fibrosis, Heart failure, Cardiac surgery, Hypereosinophilia, Cardiac magnetic resonance

## Abstract

**Background:**

Endomyocardial fibrosis is a grim disease. It is the most common restrictive cardiomyopathy worldwide, but the exact etiology and pathogenesis both remain unknown. Endomyocardial fibrosis is recurrently associated with chronic eosinophilia and probable dietary, environmental, and infectious factors, which contribute not only to the onset of the disease (an inflammatory process) but also to its progression and maintenance (endomyocardial damage and scar formation). The trademark of the disease is the fibrotic obliteration of the affected ventricle. The combination of such processes produces focal or diffuse endocardial thickening and fibrosis, which leads to restrictive physiology. Endomyocardial fibrosis affects the apices of the right and the left ventricle in around 50% of cases and most often extends to the posterior leaflet of the mitral valve. Sometimes it involves the papillary muscle and chordae tendineae, causing atrioventricular valve dysfunction. The fibrosis does not affect extracardiac organs. This cardiomyopathy is most recurrent in tropical areas of the world.

**Case presentation:**

A 67-year-old Black male with past medical history of schistosomiasis infection in childhood presented with progressive dyspnea, lower extremity edema, and weakness for 2 years. He was diagnosed with endomyocardial fibrosis. The echocardiogram showed an increased thickness in the septum (17 mm) and free left ventricular wall (15 mm), obliteration of the left ventricular apex and inflow tract, and mitral valve regurgitation. Cardiac magnetic resonance imaging revealed apical left ventricle wall thickening with left ventricular apical obliteration associated with enlargement of the respective atrium. Delayed enhancement imaging showed endomyocardium enhancement involving left ventricular apex, mitral valve regurgitation due to annulus dilation, and a thrombus at left ventricular apex. He underwent open heart surgery with mitral valve replacement, endocardial decortication, endomyocardiectomy, and two-vessel coronary artery bypass grafting as preoperative coronary angiogram showed mild right coronary artery and proximal left anterior descending artery severe lesions. Postoperative course was uncomplicated, and he was discharged successfully from the hospital. Six months after surgery, he was New York Heart Association functional class I.

**Conclusion:**

The purpose of this case report is to illustrate the aspects of endomyocardial fibrosis by reporting a case of this entity. In conclusion, progress in imaging techniques and treatment in a reference institution for cardiac diseases contribute to earlier diagnosis and survival in patients with endomyocardial fibrosis.

## Background

Endomyocardial fibrosis (EMF) is the most frequent restrictive cardiomyopathy worldwide, with higher prevalence in tropical regions of the planet. Previously known as Davies disease [1–4], it is a commonly neglected disease with insidious expression that often leads to late diagnosis and poor prognosis. The main consequences of the disease are ventricular obliteration with restrictive diastolic filling and tricuspid or mitral valve dysfunction, caused by progressive and extensive fibrosis of the ventricular endocardium. In advanced forms, EMF can lead to death, mainly by congestive heart failure or malignant arrhythmias [1, 2, 4, 5]. Only 22% of patients present symptoms [6]. While the physiopathology remains unclear, EMF is recurrently associated with chronic eosinophilia, which permits to consider endemic parasitosis (schistosomiasis, filariasis, malaria, and helminths) as a potential trigger. Reports of positive family history and higher prevalence of EMF in some ethnic groups suggest either genetic predisposition or environmental influence [2].

## Case presentation

A 67-year-old retired Black male metalworker, born and resident in Nova Lima (Minas Gerais, Brazil), was admitted to the hospital in October 2021 owing to decompensated heart failure.

He reported progressive dyspnea triggered by less than ordinary activities [New York Heart Association (NYHA) functional class III] and lower-extremity edema throughout 2 years. He denied chest pain, hospitalization due to myocardial infarction or stroke, hypertension, dyslipidemia, and diabetes. He takes enalapril 10 mg, spironolactone 25 mg, furosemide 80 mg, omeprazole 40 mg, and ferrous sulfate (40 mgFe) three tablets daily.

There was a history of schistosomiasis infection during childhood. The patient was a former smoker and alcoholic. He had a 3-pack-year history of smoking before. He had a history of daily alcohol abuse, 4 standard drinks a day, for 15 years and his last drink was 1 year ago. The family history was nonsignificant.

On physical examination, he was alert, afebrile, anicteric, and acyanotic but had mild pallor. The patient had a weight of 55 kg, height of 1.75 m, body mass index (BMI) of 18 kg/m^2^, heart rate of 82 bpm, and blood pressure of 100 × 70 mmHg. He showed no jugular venous distention. Cardiovascular examination detected a systolic murmur in mitral area and rare bilateral crackles on pulmonary basis. Examination of the other systems was unremarkable. Neurological physical examination was within normal limits. Peripheral pulses were palpable, and a +++/4+ edema was observed.

**Fig. 1:**
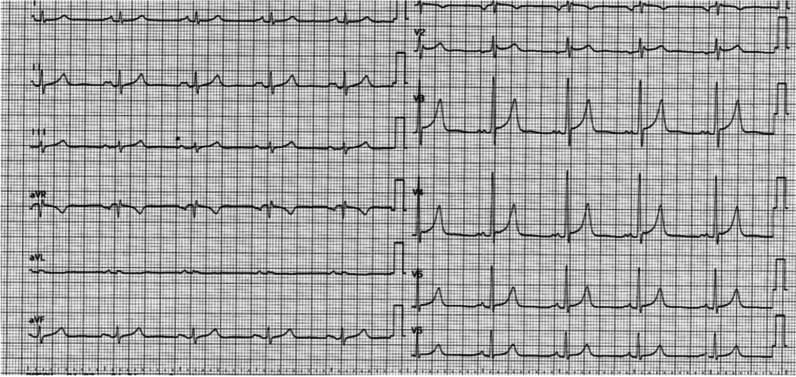
The electrocardiographic changes suggesting left atrial overload

**Fig. 2:**
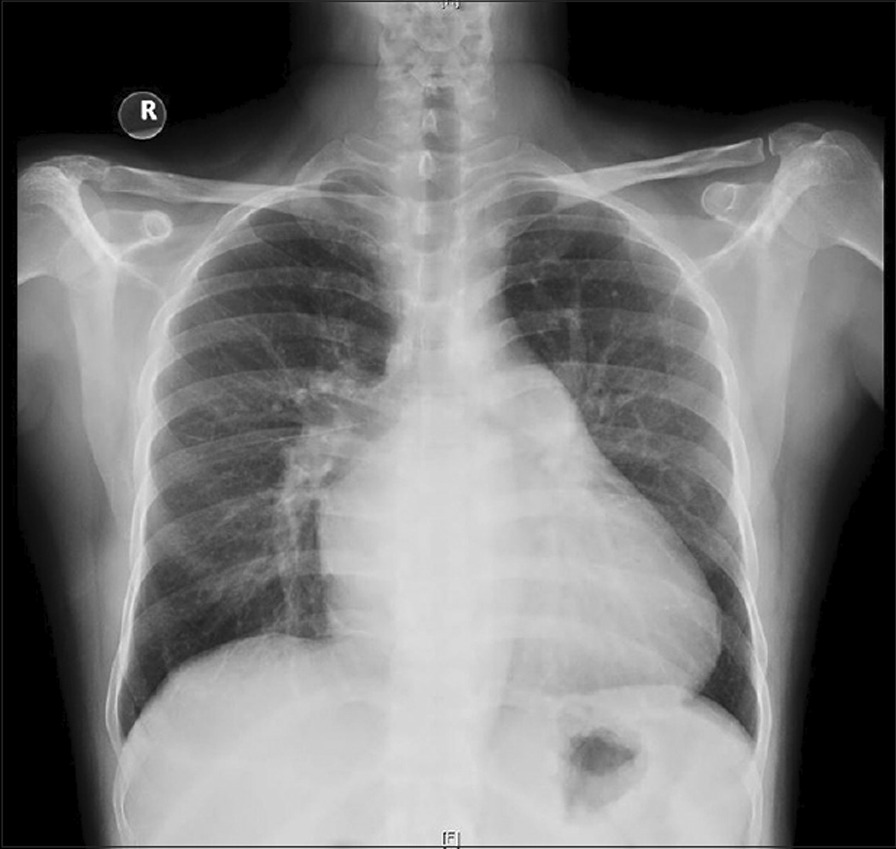
Chest radiograph, showing cardiomegaly and left atrial enlargement

Electrocardiogram (ECG) had shown a sinus rhythm, heart rate of 86 bpm, and signs of left atrial overload (Fig. 1). Cardiomegaly was seen on X-ray (Fig. 2).

Laboratory tests had shown the following results: hemoglobin 12.9 g/dL, hematocrit 39%, mean corpuscular volume (MCV) 87 fL, leukocytes 8,950/mm3 (banded neutrophils 1%, segmented neutrophils 40%, eosinophils 15%, basophils 1%, lymphocytes 34%, and monocytes 9%), platelets 210,000/mm^3^, blood glucose 84 mg/dL, urea 42 mg/dL, creatinine 1.2 mg/dL (glomerular filtration rate ≥ 60 mL/min/1.73 m^2^), sodium 133 mEq/L, potassium 4.1 mEq/L, aspartate aminotransferase (AST) 20 U/L, alanine aminotransferase (ALT) 36 U/L, uric acid 5.3 mg/dL, thyroid-stimulating hormone (TSH) 1.24 µUI/mL, free T4 (thyroxine) 1.38 ng/dL, and pro-brain natriuretic peptide (pro-BNP) 1100 pg/mL. On urinalysis, urine specific gravity was 1.007, pH was 5.5, and urine sediment had no abnormal elements.

**Fig. 3:**
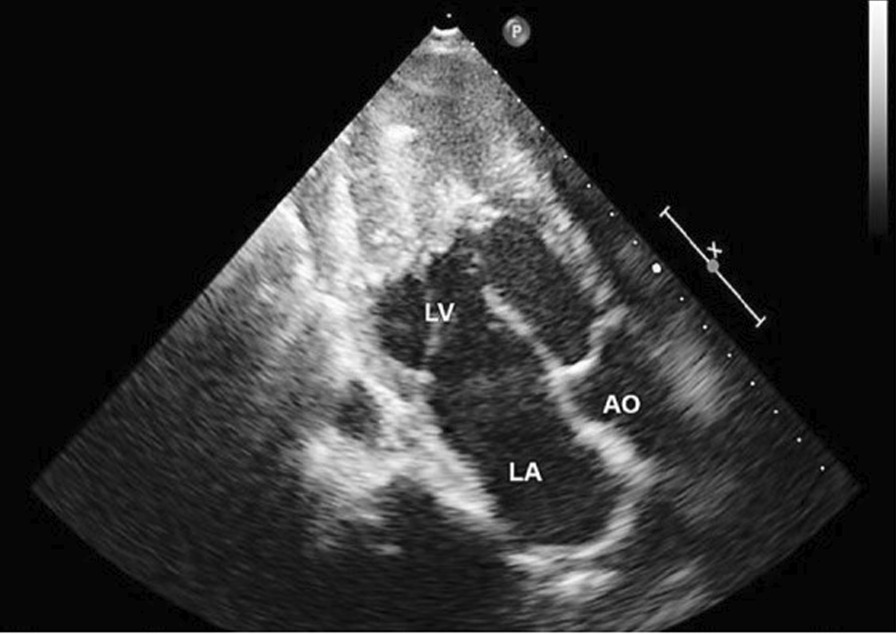
Echocardiogram revealing increased thickness in the septum (17 mm) and free left ventricular wall (15 mm), obliteration of the left ventricular apex and inflow tract, and mitral valve regurgitation. *LV* left ventricle, *LA* left atrium, *AO* aorta

The echocardiogram revealed an increased thickness in the septum (17 mm) and free left ventricular wall (15 mm), obliteration of the left ventricular apex and inflow tract, and mitral valve regurgitation; the left ventricular end diastolic pressure was 25 mmHg. Ejection fraction was normal (57%). A fibrous thrombus occupied the apex of left ventricle (Fig. 3).

**Fig. 4:**
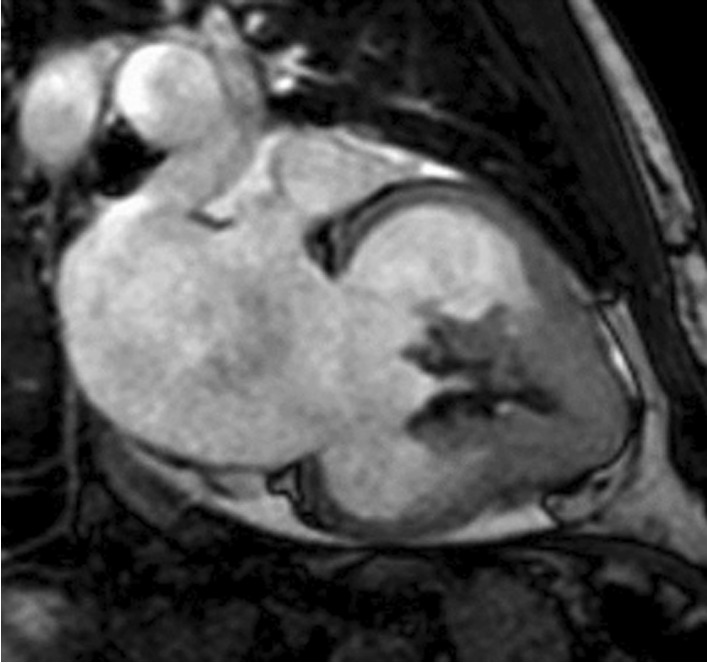
Left ventricular apical obliteration associated with enlargement of the respective atrium

**Fig. 5:**
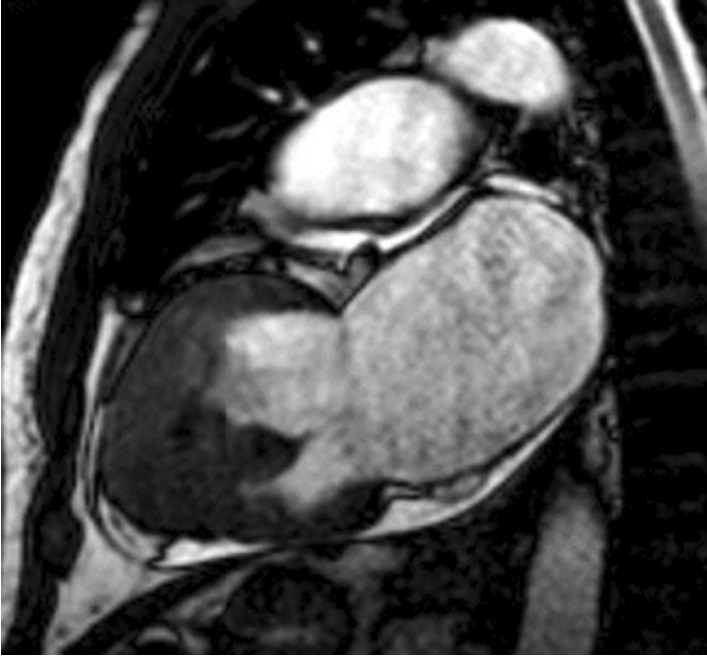
Delayed enhancement revealing endomyocardial fibrosis in the apical portions of all the left ventricle walls

Cardiac magnetic resonance imaging (MRI) revealed apical left ventricle wall thickening with left ventricular apical obliteration associated with enlargement of the respective atrium (Fig. 4). Delayed enhancement imaging showed endomyocardium enhancement involving left ventricular apex (Fig. 5), mitral valve regurgitation due to annulus dilation, and a thrombus at left ventricular apex.

The coronary angiography showed mild lesions in right coronary artery and severe lesions in proximal left anterior descending artery.

At this point, the diagnosis of left ventricle endomyocardial fibrosis (EMF), mitral valve regurgitation, thrombus at left ventricular apex, and coronary artery insufficiency was made.

He underwent open heart surgery with mitral valve replacement, endocardial decortication, endomyocardiectomy, and double coronary artery bypass grafting. After extubation, the patient was admitted to the intensive care unit (ICU) using low doses of norepinephrine and dobutamine (7 μg/kg/min). On the fifth day, norepinephrine and dobutamine administration was discontinued. ICU and hospital discharge happened on the 7th and 11th day after surgery, respectively, using beta-blocker and anticongestive therapy.

**Fig. 6:**
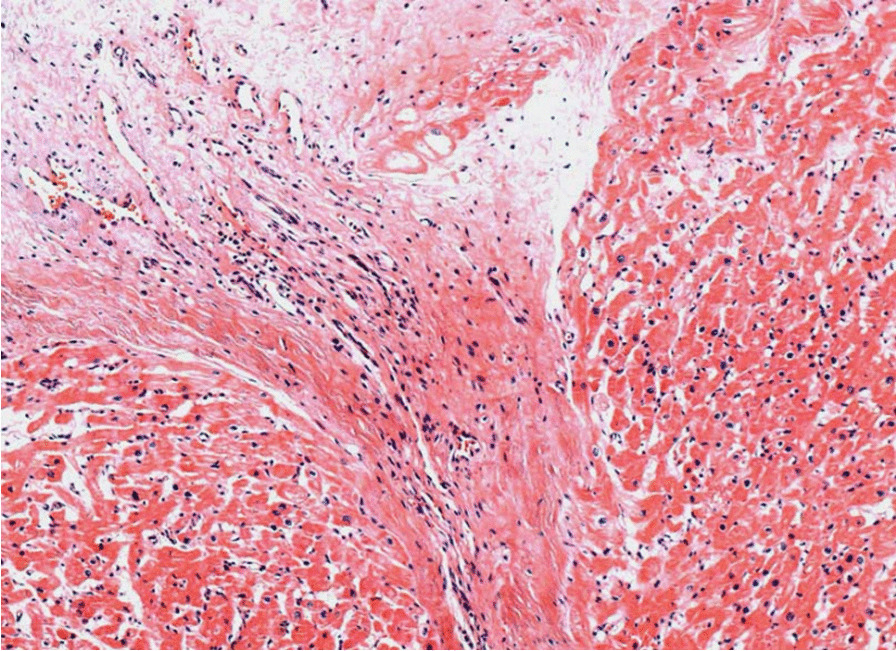
Left ventricle endomyocardial fragments showing endocardium thickening and fibrous extent into the underlying myocardium (HE, ×100)

**Fig. 7:**
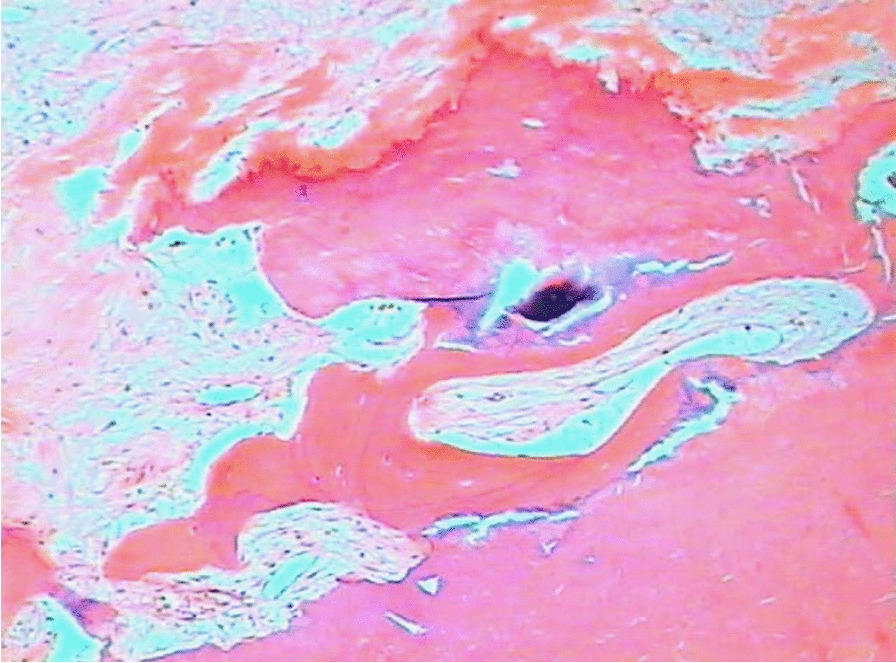
Left ventricle endocardium calcification (Hematoxylin and Eosin stain, ×100)

Tissue fragments recovered at cardiac surgery showed endocardium thickening with fibrous extent into the underlying myocardium (Fig. 6) and calcified areas of left ventricle and septum endocardium (Fig. 7). Histologically, the endomyocardium and mitral valve fragments presented areas of fibrosis, hyalinization and vascular tissue neoformation, multiple foci of mononuclear inflammatory infiltrate, and calcification areas. The biopsies were compatible with the diagnostic suspicion (endomyocardial fibrosis).

One month later, transthoracic echocardiography showed an increase in left ventricular end diastolic volume without obstruction of the left ventricle outflow tract, normal functioning prosthetic mitral valve, and a slightly decreased ejection fraction (52%).

Six months after the surgery, the patient was NYHA I. His medications included bisoprolol 5 mg per day and furosemide 20 mg per day.

## Discussion and conclusions

The case reported herein shows successful treatment of left ventricle EMF. Although EMF is a very severe disease that carries a poor spontaneous prognosis, it is possible to change the disease natural history when the treatment is performed before late complications.

EMF is the most frequent restrictive cardiomyopathy worldwide, with higher prevalence in Sub-Saharan Africa, Asia, and South America, and it is responsible for 10–20% of deaths by heart failure in Africa [1–4]. It is a chronic and insidious disease responsible for endocardial thickening, cardiac morphological modifications, reduced ventricular complacency, and heart failure. Fibrosis in the ventricular apex increases atrial size due to severe diastolic dysfunction and restrictive ventricular disorder [4, 5]. A study in Mozambique (based on a sample of 1063 individuals) estimated a prevalence of EMF of 19.8% in this rural area population. The disease affected predominantly males between 10 and 19 years old with biventricular involvement. Symptoms were present in 22% of the EMF patients [6].

Many theories have been proposed to explain the etiopathogenesis of EMF, such as viral infections, allergies associated with eosinophilia, autoimmune reactions, malnutrition, and exposure to toxic chemicals. The relationship between EMF, Loeffler syndrome (in which eosinophils gather in the pulmonary tissue in regards to a parasitic infection), and the prevalence of parasitosis in endemic countries strengthened the association between eosinophilia and myocardial lesions [2]. Approximately 40–50% of patients with eosinophilic syndrome develop heart conditions, such as Loeffler’s endocarditis, in which the hypereosinophilic reaction is responsible for fibrosis and necrosis of the endocardium, causing a endomyocardial fibrosis [7]. This theory is limited since the difference in parasitic loads is not significant when comparing the control group versus EMF patient group [2, 4, 8]. More studies are needed to confirm a strong scientific basis regarding the etiology of EMF.

Early stages usually manifest with fever, pancarditis, dyspnea, itching, and periorbital swelling [2]. Eosinophilia can be present, mainly during the early stages of EMF [9]. Abnormal increase of collagen deposition, specially type 1, and proliferation of fibroblasts cause myocardial stiffening and diastolic ventricular dysfunction [3]. If tendinous cords and papillary muscles are also affected, atrioventricular valve regurgitation occurs. Atrial fibrillation, conductive disorders, and ventricular arrhythmias are detected in the electrocardiogram in 30% of cases [5, 10]. The first necrotic stage, called the inflammatory phase, is followed by mural thrombi generation and the risk of thromboembolic complications. Endocardial calcification and valvular insufficiency are common in the final stage. This process does not involve the epicardium, and coronary artery obstruction is very uncommon. There is no involvement of extracardiac organs [11]. Symptomatic heart failure is treated with diuretics, vasodilators, and beta-blockers, depending on the disease presentation.

A score was developed by Mocumbi *et al.* in 2018 to guide the diagnosis and prognosis of patients with EMF [5, 6]. A definite diagnosis of EMF occurs in the presence of two major criteria or one major and two minor criteria [6]. The major criteria include obliteration of the right ventricular or left ventricular apex, thrombi or spontaneous contrast without severe ventricular dysfunction, retraction of the right ventricular apex, and atrioventricular valve dysfunction due to adhesion of the valvular apparatus to the ventricular wall. The minor criteria are restrictive flow pattern across mitral or tricuspid valves, pulmonary valve diastolic opening, and enlarged atrium with normal-size ventricle [6].

Diagnosis of EMF can be guided by echocardiographic evidence of thrombotic and fibrotic obliteration of the ventricular apex, increase of the endocardial diameter, and valvular regurgitation [5, 12]. Differential diagnosis versus other restrictive cardiomyopathies can be better achieved by cardiac MRI using gadolinium-based contrast. MRI provides accurate morphological evaluation by assessing diastolic and systolic functions and tissue characterization. Delayed enhancement imaging helps to identify the presence of fibrosis. It can also stratify the prognosis of most cardiomyopathies [7].

In the present case, the patient was classified as NYHA III (comfortable at rest, less than ordinary physical activity causes fatigue, dyspnea, palpitations) and had poor response to pharmacological treatment. He was in an advanced stage of the disease, and the left ventricular endomyocardium was replaced by fibrosis, as well as the mitral valve tendinous cords. The final result was a restrictive dilated cardiomyopathy and mitral valve regurgitation. Superimposed thrombosis and endocardial calcification were present. The patient underwent surgical management, with pathological confirmation of the diagnosis of EMF. Tragically, this disease still lacks a specific drug therapy. Surgical intervention is reserved only for more advanced cases (patients graded NYHA functional classification III and IV) as it has a high postoperative mortality [4, 5]. If the left ventricle is affected, the severity of the mitral regurgitation and pulmonary hypertension establish surgical indication [12]. Biventricular involvement is the most common form of the disease and is associated with a higher mortality rate. Ascites is observed in half of cases of EMF and has been associated with greater involvement of the right ventricle, longer duration of the disease, and poor prognosis. The ascites without peripheral edema appears to be caused by peritoneal inflammation [13].

Surgical intervention is expected to increase survival when performed before the development of irreversible cardiac or hepatic damage. Operative mortality is high, but successful surgery improves symptoms and seems to have a favorable effect on long-term survival [14, 15].

The progress of imaging techniques has enabled earlier detection of myocardial damage, improving patient survival in case of EMF [14]. The development of novel therapeutic agents that target eosinophils has led to a different future for patients with eosinophilic diseases. The monoclonal antibody imatinib is a promising potential treatment for EMF, but it is ineffective when heart failure is present. Finally, the diagnosis of EMF should always be considered in restrictive cardiomyopathy. There is no strong evidence of efficacy of medical treatment (diuretic, beta-blocker, or angiotensin-converting enzyme inhibitor), and surgical treatment has to be performed by experienced medical teams.

## Data Availability

All data and materials statements can be found in our institutional archives and in the bibliographic references cited in the text.

## Abbreviations

EMF: Endomyocardial fibrosis
MRI: Magnetic resonance imaging
ICU: Intensive care unit
NYHA: New York Heart Association
BMI: Body mass index
ECG: Eletrocardiogram
MCV: Mean corpuscular volume
AST: Aspartate aminotransferase
ALT: Alanine aminotransferase
TSH: Thyroid-stimulating hormone
BNP: Brain natriuretic peptide
